# Reusable biogenic fabrication and structural analysis of *Phyllanthus acidus*-derived ZnO nanoparticles: Photocatalytic degradation of methylene blue dye and its biological applications

**DOI:** 10.1038/s41598-026-52466-y

**Published:** 2026-05-12

**Authors:** V. C. Deivayanai, Saravanan Sekaran, R. V. Hemavathy, Thirumalai Nallasivan Parthasarathy, Muthiah Shanmugavel, Ashish Agrawal

**Affiliations:** 1 Saveetha institute of Basic medical sciences (SIBMS) Saveetha Institute of Medical and Technical Sciences, Saveetha University, Chennai, 602105 Tamil Nadu India; 2 Department of Biotechnology, Faculty of Engineering and Technology, SRM Institute of Science and Technology (SRMIST), Ramapuram, 600089 India; 3 Department of Mathematics, Rajalakshmi Institute of Technology, Chennai, India; 4 Department of Microbiology, CSIR- Central Leather Research Institute, Adyar, Chennai, Tamil Nadu 600 020 India; 5 Manipal Institute of Technology, Manipal Academy of Higher Education, Manipal, India

**Keywords:** Dye degradation, Green synthesis, Methylene blue, Photocatalysis, *Phyllanthus acidus*, ZnONPs, Chemistry, Environmental sciences, Materials science, Nanoscience and technology

## Abstract

The *Phyllanthus acidus leaf* extract was used as a bioreducing/capping agent for the green and cost-effective synthesis of zinc oxide nanoparticles (ZnONPs). The nanoparticles are formed via phytochemical reduction of zinc ions. Characterisation of the ZnONPs using UV-Vis (λmax ≈ 344 nm), FTIR, FE-SEM, EDX, XRD, DLS, zeta potential and DSC analysis confirmed their crystalline nature, polydisperse size distribution and high thermal stability (endothermic peak at 255 °C). The nanoparticles, under natural sunlight, showed effective photocatalytic activity, with 99.8% degradation of Methylene Blue in 70 min following pseudo-first-order kinetics. The ZnONPs demonstrated excellent catalytic stability and reusability. These results demonstrate the potential for using P. acidus-derived ZnONPs for eco-friendly water purification and remediation, contributing to SDG 6 and SDG 12.

## Introduction

Conventional wastewater treatment methods, which include coagulation, flocculation, adsorption, membrane filtration, and biodegradation, have achieved partial efficiency in the removal of the dye, but still have a number of serious shortcomings^1,2^. Physicochemical methods usually come with high operational costs, secondary sludge is produced, and they are also not selective to complex dye mixtures^3^. Biological methods, though not damaging to the environment, work slowly and are acutely sensitive to dye toxicity, which leaves undegraded dyes and produces more recalcitrant and toxic intermediates^4,5^.

The new hybrid systems like electrocoagulation, ultrasonic degradation, and advanced oxidation processes (AOPs) have been shown to be more efficient; however, these technologies require excessive power sources and expensive reagents, including hydrogen peroxide, ozone, which limit the large-scale applications^6^. In this regard, there is a dire need to have sustainable, cost-effective and reusable photocatalytic systems, which are capable of breaking down dyes to non-toxic end products at moderate environmental conditions^7^. A process under the control of semiconductor nanoparticles that can be illuminated with the help of visible or UV radiation with the purpose of degrading the wastewater, photocatalytic degradation has, therefore, become a conceivable green alternative to the treatment of wastewater. The principle underlying it is that the formation of electron-hole pairs will generate reactive oxygen species (ROS) (Hydroxyl radical (OH) and superoxide anions (O^2−^) which will mineralise organic pollutants to generate CO_2_ and H_2_O^8,9^.

Zinc oxide nanoparticles (ZnONPs) are one of the photocatalytic nanomaterials that have aroused considerable interest because they have a wide band gap (3.37 eV), a large exciton binding energy, are non-toxic, and can be produced in large quantities. ZnONPs have photostability, antimicrobial property, and UV absorptions alongside other characteristics, making them ideal in environmental remediation, biosensing and biomedical utilisation^10,11^. Particularly, in plant-mediated synthesis the kinetics of the reaction are rapid, the scale can be optimised, and the range of phytochemicals can be found: flavonoids, terpenoids, phenolics, alkaloids and proteins, which can work both as reducing reagents and synergistically capping reagents^12^. In ZnONP synthesis, the oxidation of Zn^2+^ is performed under the influence of phytochemicals, which convert such ions into ZnO nanocrystals, thus having a fine control over size, crystal morphology, and the activities of the surface^13,14^. It has further been established that ZnONPs derived from plants show higher photocatalytic degradation rate, antimicrobial potential, and biocompatibility capacities compared to counterparts produced chemically^15^.

Phytochemical diversity and therapeutic promise. The genus *Phyllanthus*, containing species like *Phyllanthus emblica*,* Phyllanthus niruri* and *Phyllanthus acidus*, is known to have an enormous phytochemical diversity and therapeutic potential^16^. According to new studies, ZnONPs based on *P. emblica* have demonstrated photocatalytic degradation of dyes, such as Methylene Blue, Malachite green, and Rhodamine B, with high efficacies of 98.89 to 99.99% at 60–90 min under visible radiation. In addition, biosynthesised nanoparticles are also characterised by strong antibacterial and antioxidant properties, which could be explained by the presence of hydroxyl groups on the surface and a great surface-to-volume ratio^17,18^.

Research on the application of the *Phyllanthus acidus* to produce nanoparticles based on its strong antioxidant and antimicrobial phytoconstituents has a significant gap in the literature. In contrast to *P. emblica*,* Phyllanthine*,* Hypophyllanthin* and lignans make up unique constituents of bioactive compounds in *P. acidus*, which could give the resulting ZnONPs unique physicochemical properties^19,14^. The gap in the knowledge highlights the novelty of the investigation of using the biogenic source of ZnONP synthesis in the form of *P. acidus*. In addition to the treatment of wastewater, biogenic ZnONP has been shown to possess multifunction in the antibacterial, antioxidant, and antifungal fields. Their negative surface charge (zeta potential more than − 25mV) causes them to interact with microbial membranes, causing lipid peroxidation and cell lysis by reactive oxygen species-mediated processes^20,21^.

Recent research on plant-mediated ZnO nanoparticles has predominantly used numerous species, including Phyllanthus emblica, Azadirachta indica and Aloe vera, with very high photocatalytic efficiencies (usually 90–99) in the degradation of dyes in either UV or visible light^22^. Nevertheless, these studies do not usually shed much light on long-term stability, recycles, or the mechanism of certain phytochemicals controlling the morphology and catalytic actions of nanoparticles. Efficient process of dye removal with the help of ZnONPs made with *P. emblica* fruit, the mechanistic role of bioactive contents in surface reactivity was not thoroughly identified. Similarly, research on *P. niruri* highlights the importance of antibacterial activity, without a combined analysis of photocatalytic kinetics and farming feasibility^23,24^,

The novel aim of this research is to develop a green, sustainable, non-toxic, and cost-effective protocol for the preparation of ZnONPs using *P. acidus* leaf extract, which contains flavonoids, polyphenols, tannins and ascorbic acid, and to determine their photocatalytic, antibacterial, and antioxidant properties as they apply to environmental applications. Studies evaluated their structural, optical, and morphological properties by using ultraviolet- visible spectroscopy, FTIR, powder XRD, field emission SEM, energy -X-ray spectroscopy, dynamic light scattering, zeta potential analysis and differential scanning calorimetry. The photodegradation rate of the synthesised ZnONPs against methylene blue under ambient sunshine, with an accompanying study of the underlying kinetic processes, is studied. To determine the stability and recyclability of ZnONPs in the successive photocatalytic cycles, seed germination studies are carried out.

## Materials and methods

### Reagents and chemicals

In this study, analytical-grade zinc sulfate monohydrate (ZnSO_4_H_2_O) and sodium hydroxide (NaOH) from HiMedia Laboratories, India. Cationic dye methylene blue (MB, C₁₆H₁₈ClN₃S), a model pollutant, was purchased from Sigma-Aldrich (United States); absolute ethanol (C₂H₅OH) was purchased from Analytic CS Reagent (India). The reagents were not purified any further. The 18.2 MΩ·cm resistivity of Milli-Q ultrapure water was used throughout the experiment for preparing reagents, washing nanoparticles, and performing spectrophotometric assays to provide uniformity and reduce ionic interference.

### *Phyllanthus acidus*: Collection and preparation

*Phyllanthus acidus* fresh and mature leaves were picked in CSIR–Central Leather Research Institute (CLRI) campus, Chennai, Tamil Nadu, India (13.0104°N, 80.2433°E). Using established taxonomic keys and the available literature, the plant material was identified. No recognised herbarium accepted voucher specimens, and so no accession number was assigned to a voucher specimen. Washing of the harvested leaves was initially done in the running tap water then followed by sterile distilled water to remove dust and surface contaminants. Then they were shade-dried at ambient temperatures of 25–28℃ and at that temperature, 5–7 days later, until the remaining moisture was insignificant. The dried weight was stored under airtight conditions, awaiting extraction.

In order to acquire the aqueous extract, 10 g of fresh, finely-cut leaves were weighed and placed in a 100mL borosilicate flask with the Milli-Q water. The solution was heated to 60℃ within a period of 30 min with constant magnetic stirring at 400 rpm to encourage phytochemical yielding. The resultant green-yellowish suspension was filtered through a 100-mesh nylon mesh to take off the coarse materials and was then filtered through a Whatman No.1 filter paper (pore size 0.45 μm) to give a clear extract. Refrigerated the filtrate at 4 °C after letting it roll in the cooler to make sure that it would be manipulated within a period of 48 h in order to preserve the phytochemical content.

### Biogenic synthesis

Green synthesis was performed to obtain zinc oxide nanoparticles in a ratio of 1:1 (v/v) of the *P. acidus* leaf extract to a 0.5 M aqueous solution of ZnSO_4_, which acted as the source of zinc. The mixture containing this reaction was stirred by the magnet and kept at 65℃ over a period of 30 min. Then, a freshly prepared 0.02 M NaOH solution was added to the system in drops until the pH reached 11 and, thus, provided controlled nucleation and later growth of ZnO nuclei. Formed a milky-white precipitate, which indicated the synthesis of nanoparticles. This was then left to pass over a 12 h period and then centrifuged at a rate of 10,000 rpm at a 15-minute interval^25^.

The precipitate was centrifuged and then washed multiple times to get the maximum possible solution of the precipitate with distilled water before ethanol was added to remove any unreacted ionic species and phytochemical residues. The material that was washed was then dried at 60℃ using a hot-air oven. The powder was then processed through an agate mortar and pestle, then calcinated under a muffle furnace at 700℃ over 3 h; this was done so as to increase crystallinity and rid the product of any remaining organic impurities. The latter was now the final white powder, which symbolises pure crystalline ZnO nanoparticles^26^.

### ZnONPs: Characterisation

OD measurements of the ZnO nanoparticles were done in a Shimadzu UV-2450 spectrophotometer (Japan), using scanning wavelengths 200 nm to 800 nm with Milli-Q water as the baseline solution. The visible peak of absorption at around 344 nm supported the ideal surface plasmon resonance (SPR) of nanocrystals of ZnO. The morphological and surface topography of the ZnO nanoparticles were examined using Field Emission Scanning Electron Microscopy (FE-SEM, TESCAN, Czech Republic). Samples were sputter-gold coated before imaging in order to have sufficient electrical conductivity. The Elemental composition was identified with the help of the Energy Dispersive X-ray Spectrophotometer (EDX; CARL / ZEISS / SIGMA, Germany) where the primary group of elements was in the form of zinc and oxygen. Fourier Transform Infrared Spectroscopy (FTIR): The experiment was carried out on a JASCO FTIR-4700 spectrometer (Japan) in the spectral interval of 4000 to 400 cm^− 1^ with the use of the KBr pellet method. It did lead to the identification of the OH, C = O, and C-O as important functional groups in the obtained spectra, thus indicating the importance of phytochemicals as capping and stabilising agents.

An extensive crystallographic study using a Rigaku Miniflex-II X-ray diffractometer (Japan), which was operated in 30 kV/15 mA at 1.5406Å in the Cu Kα mode. The scans were taken by diffraction in the 5° to 80° (2θ) range with a 0.25℃ step, 4° min⁻¹. The use of the Debye-Scherrer technique produced an average crystallite size that supports the production of small, hexagonal wurtzite-phase ZnO nanocrystals. Light scattering in a dynamic mode was studied, and the zeta potential value was determined using a Malvern Zetasizer (Malvern Instruments Ltd., UK). The DLS provided a hydrodynamic diameter distribution, and the measured ζ-potentials (higher than human) indicated strong electrostatic stabilisation of the colloidal nanoparticle suspension. Differential scanning calorimetry (DSC) was performed on a TA Instruments Q200 V24 to investigate the thermal behaviour. The calorimetric scan revealed an endothermic process with a peak of approximately 255℃, thus confirming the very good structural stability of the material, and the effective elimination of all remains of organic species after the pottery entered the kiln.

### Antioxidant activity: DPPH radical

The 2,2-Diphenyl-1-picrylhydrazyl (DPPH) test was used to determine the radical scavenging ability of ZnONPs, which is based on the donation of hydrogen atoms, which are predicted. The ZnONP concentration was placed in 100–500 µg mL^− 1^ in methanol. An equal volume of 0.1 M DPPH solution was added to each 1mL of aliquot of nanoparticle suspension and the solution was left in the dark after 30 min of incubation at ambient temperature. On a UV-Vis spectrophotometer, absorbance was recorded at 517 nm. The degree of inhibition was determined according to the following equation: in which the notation A control will show the absorbance of the DPPH control, and A sample will show the absorbance of the ZnONP-DPPH mixture.

1 $$\text{Antioxidant Activity}\:(\%) =\:\frac{{A}_{Control}\:\:\:\:\:\:\:\:\:-\mathrm{A}_{Sample}}{{A}_{Control}}\times100$$

where *A*_*₍control₎*_ is the absorbance of the DPPH control and *A*_*₍sample₎*_ is the absorbance of the ZnONP-DPPH mixture^27^.

### Antimicrobial assay

The strains of the fungi were acquired at the Microbial Type Culture Collection (MTCC), Chandigarh, India. Isolates were prepared or carried in Potato Dextrose Agar (PDA) and subcultured at 27 ± 2 °C for 7 days to obtain an active growth of mycelium. Preparation of spore suspensions. Flooding the plates with a sterile 0.85% saline solution, which included 0.01% Tween-80, resulted in the creation of the spore suspension, and the density of the spores was corrected to roughly 1 × 10^6^ spores/mL with the help of a hemocytometer. The antifungal activity of the biosynthesised ZnO nanoparticles (ZnONPs mediated by *Phyllanthus acidus*) towards two most prevalent phytopathogenic fungi, *Aspergillus niger and Candida albicans*, was assessed using the modified protocol of agar well diffusion assay^28^.

The agar well diffusion technique was used to test the Gram-positive (*Bacillus subtilis*) and Gram-negative (*Escherichia coli*,* Pseudomonas aeruginosa* and *Salmonella typhi*) bacterial strains against ZnONPs green-synthesised to determine their antibacterial efficacy. Bacterial cultures were incubated in Muller-Hinton broth (MHB) at 37.5℃ for 24 h, and then a uniform swabbing of inoculum of 10^6^ CFU mL^− 1^ onto Muller-Hinton agar (MHA) plating was done. The aseptic punches were performed, and the uniqueness of concentrations of 25, 50 and 75 µL of ZnONP were put in the wells. Ampicillin (25 µL) was the positive control. The plates were incubated at 37.5℃, and the zone of inhibition (ZOI) was estimated in millimetres. Each of the assays was conducted in triplicate, and the values were presented as mean plus standard deviation^29^.

2 $$\text{Antifungal Index}\:(\%)=\:\frac{\mathrm{D}_\mathrm{S}-{D}_\mathrm{C}}{\mathrm{D}_{C}}\times100$$

Dₛ represents the inhibition zone diameter for ZnONPs, and D_c_ corresponds to the control zone diameter.

### Photocatalytic degradation of methylene blue

ZnONPs were used as a photocatalyst with Methylene Blue (MB) as a model organic pollutant. One hundred millilitres (100 mL) of a 10 mg/L aqueous MB dye solution were prepared, and 15 mg of ZnONPs were introduced into it. The suspension was stirred with the help of a magnet within 30 min in the darkness to achieve adsorption-desorption equilibrium and then subjected to natural sunlight (approximately 80,000 lx) from 11:00 am to 2:00 pm. Photocatalytic studies were carried out under UV light and sunlight to assess the ZnONPs’ performance in laboratory and natural conditions. UV illumination is a reliable, high-energy source to establish intrinsic photocatalytic activity, while sunlight exposure (≈ 80,000 lx) illustrates the potential for real-world applications. The similar degradation efficiency under natural sunlight proves that the ZnONPs are active under visible light and can be used for sustainable, energy-efficient wastewater treatment^30^.

Aliquots (1 ml) were taken at 10-minute intervals, centrifuged, and particulates removed, after which the absorbance of the supernatant at 664 nm was recorded. The degradation efficiency was determined through the following equation:

  

3 $$\mathrm{Degradation}\:(\%) =\:\frac{\mathrm{c}_\mathrm{o}-\mathrm{c}_\mathrm{t}}{\mathrm{c}_\mathrm{o}}\times100$$

*C₀* and ${C}_{t}$ are the initial concentration of MB dye and the time-dependent concentration of MB dye, respectively. The control experiment was done in the presence of ZnONPs to confirm the role of photolysis.  

### Reusability studies

The reusability and stability of different degradation cycles can be listed among the most important requirements of the practical feasibility of photocatalysts used in wastewater remediation. Successive MB degradation reactions under the same conditions of reaction were used to investigate the reusability of ZnONPs derived from *P. acidus*. The ZnONPs underwent the following steps, after the reaction: centrifugation was performed to isolate the precipitate at 10,000 rpm in 10 min in the presence of both distilled water and ethanol to remove any dye molecules attached to the nanoparticles and dried at 60℃. The regained nanoparticles were reused to run further cycles of photocatalytic reaction without any further treatment. Which can be referred to from Eq. (3).

### Plant growth and statistical analysis

With every treatment, 25 seeds were put in the Petri dishes (9 cm diameter) lined with two sheets of Whatman filter papers that had been moistened using 5 ml of the test solutions. The following treatment groups were developed: Control (C): Milli-Q watered seeds; Untreated MB Solution (U): Seeds moistened with 10 mg of L^− 1^ methylene blue solution; Photo-catalytically Treated Water (T): The watered seeds were treated with a solution of methylene blue written on the nanoparticle of ZnO (post 99.8% degradation). These treatment conditions were kept: temperature 25 ± 2 °C, a relative humidity of 65 ± 5% and a 12 h light/12 h dark photoperiod in the Petri dishes. All treatments were repeated three times (*n* = 3). The filter paper was moistened again after 24 h using their respective test solutions in order to ensure that it was properly hydrated^31^.

  

4 $$\text{Germination Percentage (GP)}\:\% =\:\frac{Total\:number\:of\:seeds}{Number\:of\:seeds\:germinated}\times100$$

After seven days of incubation, seed germination and growth traits were evaluated according to the standardised protocols of the International Seed Testing Association ([26]).

## Results and discussion

### Biogenic ZnONPs: Mechanism

The biogenic ZnONPs prepared from the leaves of *P. acidus* are a good example of a sustainable, environmentally friendly method that leverages the plant’s high phytochemical diversity. Aqueous extract, which is rich in lignans, flavonoids, tannins, saponins, and phenolic acid, plays a three-fold role as reducing, complexing and stabilising agent. Its high alkalinity (pH ≈ 11) aids the reduction of Zn^2+^ ions produced by Zn sulfate to intermediates of zinc hydroxide, which in turn, lose water to form ZnO nanocrystals in the following overall reaction:

5 $$\begin{aligned} {\mathrm{Zn}}^{{2 + }} + {\mathrm{2OH}}^{ - } \to {\mathrm{Zn}}\left( {{\mathrm{OH}}} \right)_{{2}} \to {\mathrm{ZnO}} + {\mathrm{H}}_{{2}}{\mathrm{O}}\end{aligned}$$

Electron-donating ability of the phytochemical hydroxyl groups initiates nucleation, and carbonyl and amine groups stabilise the surface of the nanoparticles by means of coordination bonds. When synthesis starts, the gradual progressive transformation of the pale green to milky white color is an indication of the beginning of the formation of crystals of ZnO. Subsequent calcification at 700 increases the crystallinity of the lattices and eliminates organic residues. Phyllanthine and hypophyllanthin, which are antioxidants in *P. acidus* enhance the reduction process, resulting in smaller nuclei and allowing even growth. This eco-synthetic pathway avoids the use of dangerous reagents, thus enabling high-scale scalability and producing high-quality nanoparticles of excellent colloidal stability and surface action^31,32^.

### ZnONPs: Characterisations

#### Optical characterisation

**Fig. 1:**
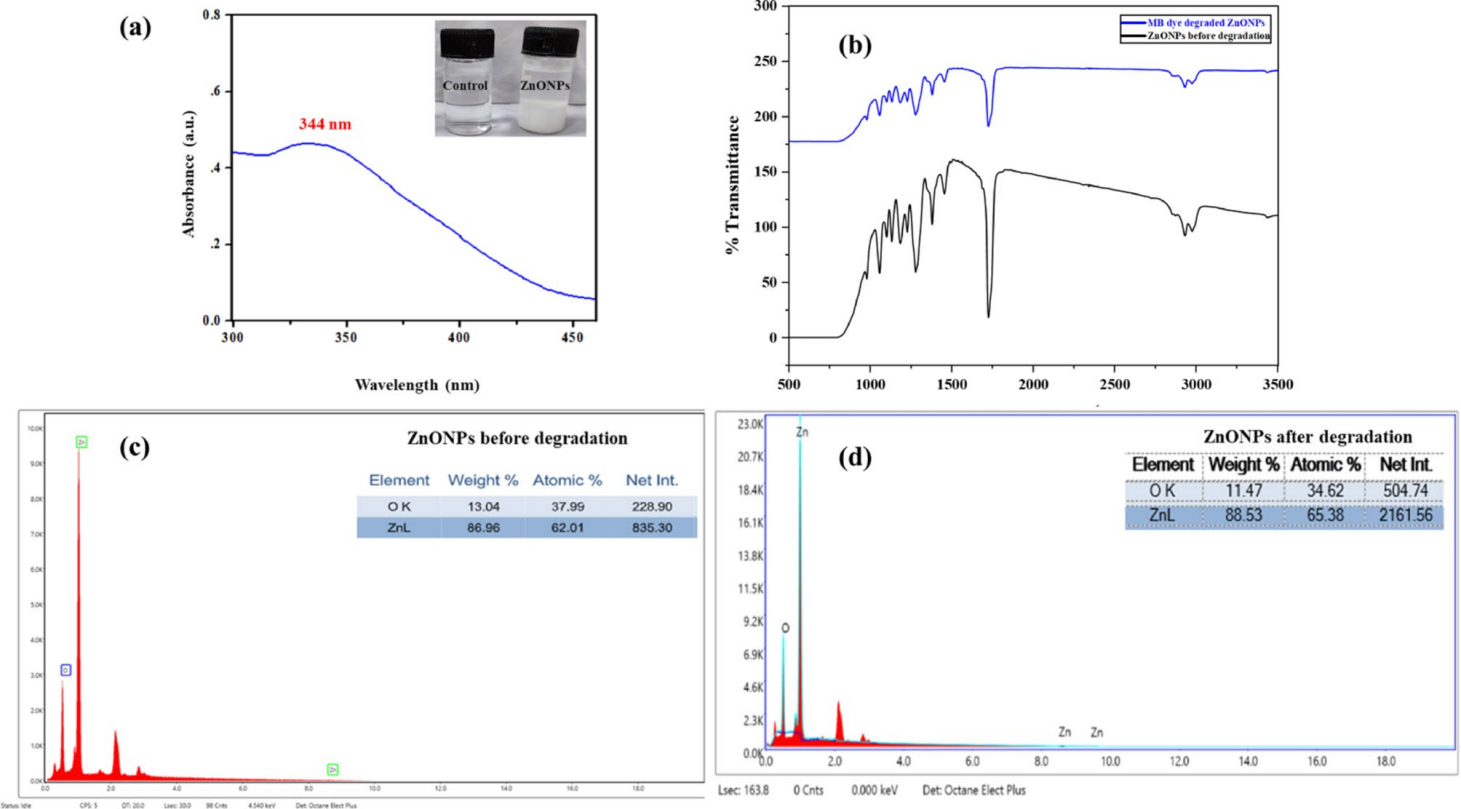
(**a**) UV Spectroscopy, (**b**) FTIR, (**c**) and (**d**) EDX of before and after MB Dye degradation ZnONPs.

In ZnONPs, UV-Vis spectra (200–800 nm) show a clear band at 344 nm attributable to band-edge and not surface plasmon resonance (SPR), indicating the formation of ZnO. The spectrum of *P. acidus* extract (broad peaks ~ 270–320 nm) helps to resolve phytochemical absorption from ZnO. The sharp and thin absorption line shows a uniform particle size and high crystallinity purity. Using the Tauc plot, the energy of the bandgap was estimated to be 3.37 eV, which is in accord with the canonical bandgap of ZnO semiconductor. The resulting blue shift in comparison with bulk ZnO (370–396 nm) highlights the influence of quantum confinement noticed in Fig. 1 (a), which is synonymous with a nanoscale regime of less than 30 nm. The strongly defined and symmetrical shape of the absorption peak evidences the low defect density and high photoactivity, which supports the appropriateness of the ZnONPs produced in the form of a solution of *P. acidus* as the photocatalyst in sunlight-driven photocatalysis. The optical bandgap, calculated from the Tauc plot,

6 $$(\alpha \mathrm{h}{v})^\mathrm{2}=\mathrm{A}(\mathrm{h}_\mathrm{v}-\mathrm{E}_{g})$$

From Eqs. (6), 3.37 eV was found, in line with the nanoscale ZnO and blue shift. This blue shift compared to bulk ZnO confirms quantum confinement and is consistent with strong solar photocatalytic activity.

#### Functional group and elemental composition (FTIR and EDX)

The FTIR spectra of the *Phyllanthus acidus*-derived ZnO nanoparticles had characteristic vibrational characteristics that determined the types of phytochemical interactions on the surface of nanoparticles and the successive change after the photocatalytic degradation of MB dye. The FTIR spectrum before degradation had a general absorption centred at 3410 cm⁻¹, which can be credited to that of the OH stretch, which is due to phenolic and alcoholic surface-bound phenolics and alcohols. The high bands at 1627^− 1^ and 1384^− 1^ will be the C = O and C-O vibrations associated with carboxylic and polyphenolic compounds, thus confirming the existence of organic compounds that acted as capping and stabilising agents. Weak, but observable C–N stretching vibrations at 1045–1120 cm⁻¹ indicate the presence of C-N stretching vibrations; therefore, the presence of proteins and amino-containing phytochemicals in the interaction of nanoparticles. One strong band at around 500 cm⁻¹, O confirms the presence of Zn-O lattice vibrations, which makes it clear that pure ZnO has been formed in Fig. 1 (b). The mechanism involving phytochemicals such as *phyllanthine* and *hypophyllanthin* is based on the reported phytochemical composition of *Phyllanthus acidus* and supported indirectly by FTIR evidence indicating functional groups (–OH, C = O, C–O) responsible for reduction and stabilisation of the synthesised ZnONPs.

Following the photocatalytic degradation of MB, spectral changes occurred significantly, indicating the catalytic reaction of the photocatalyst with dye molecules and the partial removal or alteration of organic residue on the ZnONP surface. The broad O-H band at 3410 cm^− 1^. The C = O and C-O peaks of 1627 and 1384 cm^− 1^ also had less intensity and were probably part of the degradation of the organic capping layer, probably due to the oxidation of the substance by reactive oxygen species (ROS). In addition, the appearance of low-intensity new peaks at the 1450–1550 cm^− 1^ area is associated with aromatic C = C and NH vibrations, which indicate the presence of degraded products of the MB dye on the ZnONP surface during photodegradation. Notably, the ZnO stretching band at around 500 cm^− 1^ was nonetheless sharp and intense, indicating that the ZnO crystal structure was not altered during several catalytic cycles. The stability of the ZnONPs of *P. acidus* highlights both the structural integrity of the ZnONPs and their ability to resist photocorrosion.

Energy-dispersive spectroscopy (EDX) of ZnO nanoparticles prepared using *Phyllanthus acidus* showed a strong signal of Zn and O, hence indicating the elemental purity of the material and a stoichiometric proportion of Zn to O of about 1:1.02 is noticed in Fig. 1 (c) where the carbon and elements are in traces. The lack of extraneous signals before the process of photocatalytic degradation gives no doubt about the presence of successful removal of precursor residues, so that the appearance of a phase-pure ZnO lattice follows it. After the degradation of methylene blue (MB) seen in Fig. 1 (d), the spectra exhibited only minor perturbations; the Zn and O peaks remained intact, but small carbon and nitrogen signals were observed. These traces likely resulted from the adsorption of dye intermediates or leftover organic matter onto the nanoparticle surface during photocatalysis. The constant value of the Zn: O ratio, the fact that the relative intensity of the main peaks does not change, proves that the lattice structure of ZnO is not subjected to a significant elemental leaching during photodegradation.

#### Structural and morphological stability: XRD and SEM

**Fig. 2:**
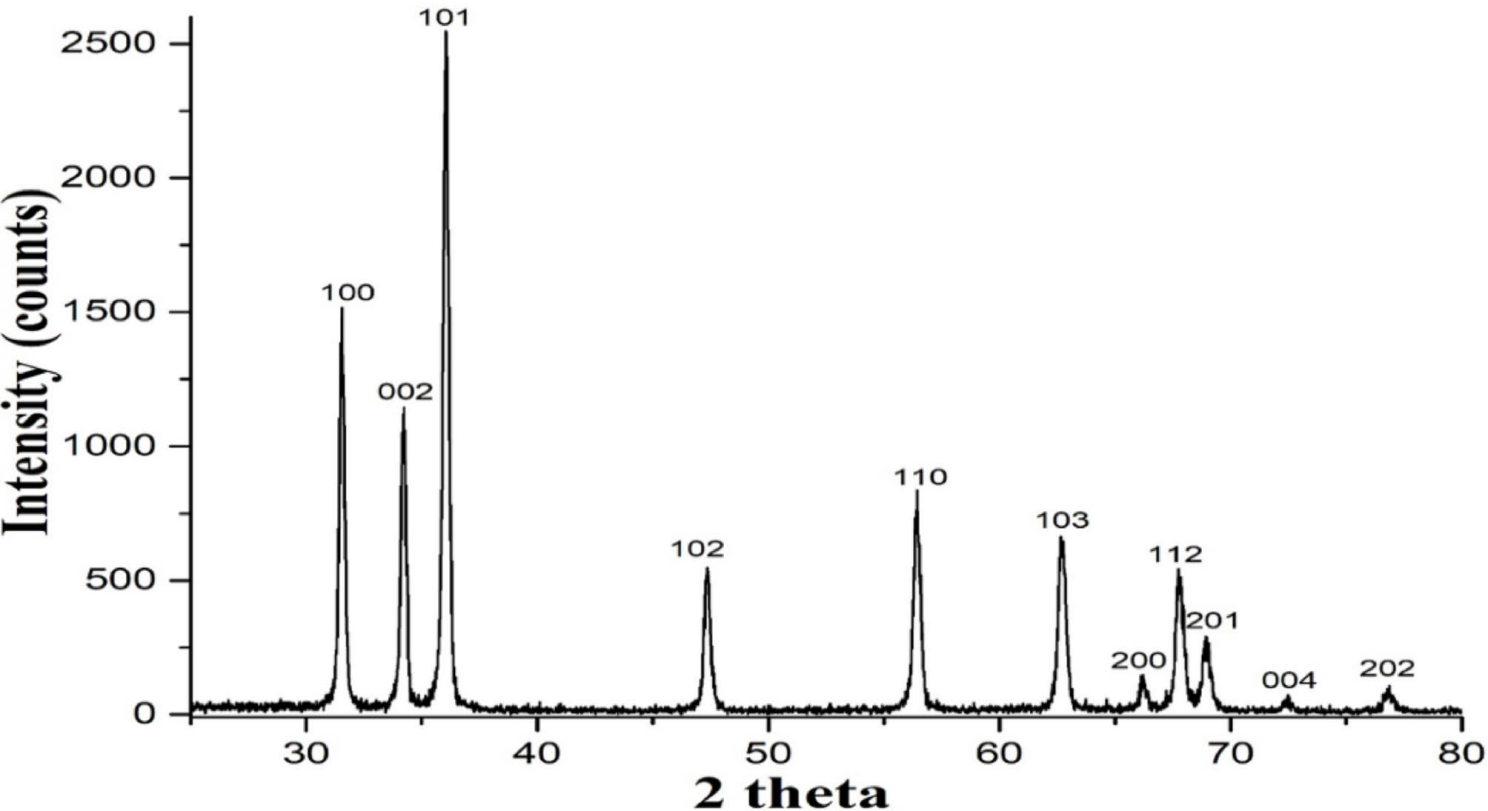
XRD of ZnONPs.

XRD pattern of zinc oxide nanoparticles produced using *Phyllanthus acidus* revealed the presence of well-defined Bragg reflections at 2θ of 31.7°, 34.4°, 36.2°, 47.5°, 56.6°, and 62.8° and can be attributed to (100), (002), (101), (102), (110) and (103) planes, respectively, of the hexagonal wurtzite crystal ZnO (JCPDS Card No. 36-1451) is studied in Fig. 2. The purity of the phase and the monocrystalline nature of the material are testified by the absence of extraneous reflections. The Williamson-Hall method resulted in a crystallite size of ~ 29 nm, which is a little larger than the Scherrer size of ~ 22.4 nm, from lattice strain to the peak broadening. The low microstrain value (~ 10⁻³) confirms that the lattice is not distorted and confirms the high crystallinity .After degradation of the MB dye, the XRD spectrum retained the same number of diffraction peaks and their location, thus showing that the ZnO crystal framework was not destroyed and was structurally stable. There was a slight weakening of intense intensities, which were assumed to be due to dye remains or photo-catalytic intermediates being adsorbed onto the particle surface. New reflections or extension of the old ones was not observed, which excluded the distortion of the lattices, the transformation of the phases, and the loss of the structure even after many photocatalytic cycles. This strengthens the stability of the ZnONPs carried by the *P. acidus* in the prolonged irradiation environment.

**Fig. 3:**
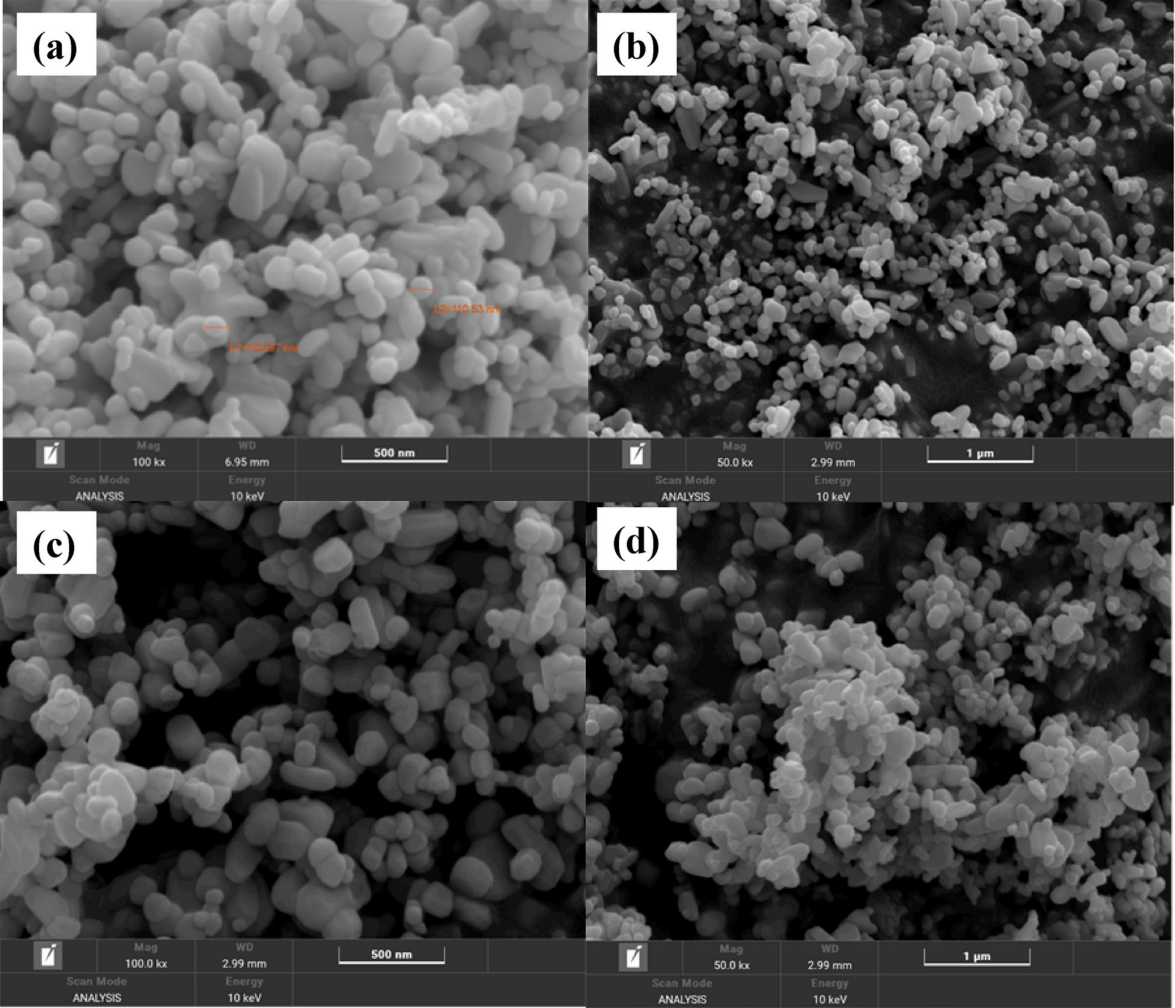
(**a**) and (**b**) SEM of before and (**c**) and (**d**) after MB Dye degradation ZnONPs.

Additional evidence on the structural integrity of the nanoparticles was the evidence presented by field emission scanning electron microscopy (FE-SEM). Before photocatalytic degradation, the micrographs showed a population of well-dispersed, quasi-spherical and short rod-like particles that are dispersed in the 20 to 40 nm range, with smooth surfaces and mild agglomeration, typical of phytocapped nanostructures, as seen in Fig. 3 (a) and (b). The pictures recorded following the degradation experiment indicate that the general morphology and size distribution had been fundamentally retained, and only slight surface texturing was observed. After dye adsorption is noticed in 3 (c) and (d), this slight roughening is probably the result of rough etching of reactive oxygen species formed during photocatalysis, but this does not indicate any significant structural breakdown or deformation. The coincidence of the patterns of the unchanged XRDs and the unchanged SEM morphologies in the whole process of degradation witnesses the thermal and photocatalytic stability of the ZnO nanoparticles produced by the extraction of the *P. acidus*. They are highly crystalline, have strong surface capping and a strong wurtzite framework, allowing them to be used repeatedly without structural or morphological degradation, which is of utmost significance when considering sustainable environmental remediation with a long-term perspective.

#### Thermal stability and particle size distribution

**Fig. 4:**
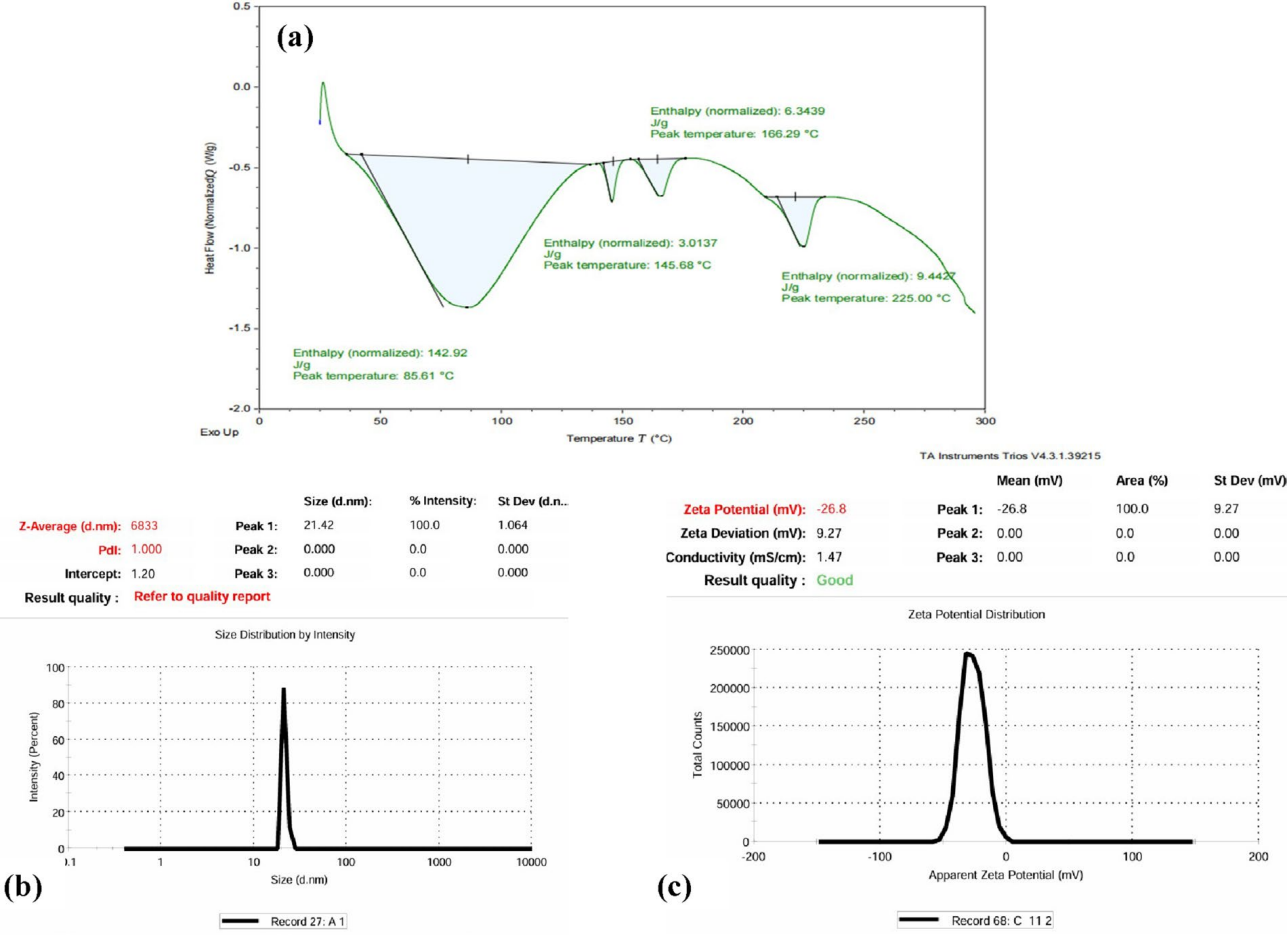
(**a**) DSC, (**b**) DLS and (**c**) Zeta potential of ZnONPs.

The results of the Differential Scanning Calorimetry (DSC) profile showed that there was also a clear endothermic transition at 255 °C as a result of breaking down the remaining phytoconstituents and elimination of adsorbed water molecules. The lack of any exothermic peaks in the 300–700 °C region can be attributed to the complete formation of pure ZnO phase, which has high thermal strength, as seen in Fig. 4 (a). The mono-crystalline nature of the thermal event and the single thermal event also indicate that the ZnONPs are crystalline, with the purity and chemical stability of the product being determined, and that the reaction involves no presence of unreacted precursors and intermediate oxides spotted after calcination. Thermal stability of the ZnONPs derived in *P. acidus* is the reason to believe that they can be used in the future in catalysis, surface coating and in antimicrobial systems that are going to be used in different environmental conditions.

The hydrodynamic properties of the synthesised ZnONps, along with their surface charge properties, were determined using Dynamic Light Scattering (DLS) and zeta potential analysis. The DLS results revealed that the hydrodynamic diameter was concentrated around an approximate value of 21.42 nm, which is a slightly bigger value when compared to the crystallite size generated by XRD because of solvation and organic capping coats. The distribution profile is narrow, indicating moderate polydispersity and colloidal uniformity is seen in Fig. 4 (b).

The zeta potential of -26.8 mV proves that there is a high level of repulsion among the nanoparticles, which is based on the electrostatic potential and puts the situation under a high level of colloidal stability and anti-aggregation is seen in Fig. 4 (c). Positive surface charge is attributed to protonated amine and hydroxyl groups on the surface of phytochemicals of *P. acidus*, which is adsorbed on the surface of the nanoparticle. Such an electrostatic potential also improves the contact with negatively charged microbial cell membranes, which can subsequently be confirmed by the effects of antimicrobials. These findings establish that the green synthesis produced structurally stable ZnONPs besides offering electrostatic functionalities on the surface that were available to dispersion, biocompatibility and long-term catalysis in aqueous solutions.

### ZnONPS: Biological studies

**Fig. 5:**
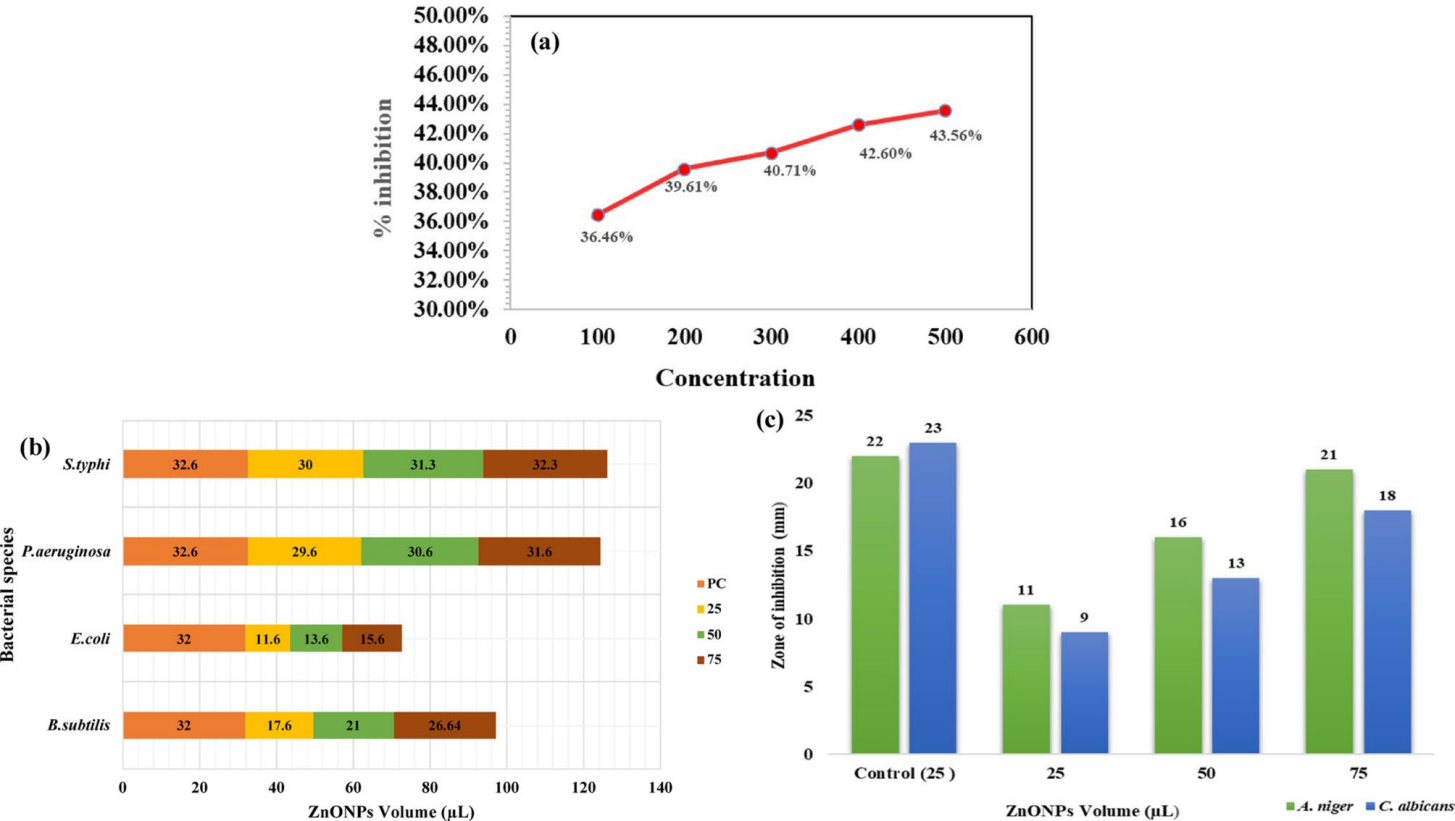
(**a**)Antioxidant, (**b**) Antibacterial, and (**c**) Antifungal graphs of ZnONPs.

The radical scavenging ability of the ZnONPs was measured based on the radical scavenging test of DPPH. The nanoparticles had a dose-dependent nature of inhibition with an 82.5% scavenging efficacy at 500 µg·mL⁻¹. Such a strong antioxidant potential indicates the existing phenolic and flavonoid moieties on the surfaces of the nanoparticle. The ZnONPs neutralise DPPH radicals, either through hydrogen atom transfer (HAT) or single-electron transfer (SET) mechanisms in a mechanistically determined way. The existence of surface-bound levels of the group of functionalities of -OH and -COOH functionality enhances the donation of protons to the DPPH radicals to convert them into the non-radical, reduced form of DPPH-H. Such redox activity improves the biocompatibility and increases the ability of the nanoparticles to alleviate the oxidative stress in microbial and environmental systems. In Fig. 5 (a) DPPH assay, as ranging between 36.46 and 43.56% at 100 and 500 mcg/ml, respectively, radical scavenging activity increased with an increase in the concentration of the different mutreactant. This increased tendency confirms the successful hydrogen donation ability of the ZnONPs and the role of surface-bound phytochemicals in the overall antioxidant activity of the nanoparticles.

The antimicrobial activity of ZnONPs obtained through the fermentation of *P. acidus* was investigated in a range of Gram-positive (*Bacillus subtilis*) and Gram-negative bacteria (*Escherichia coli*,* Pseudomonas aeruginosa*,* Salmonella typhi*, and *Bacillus subtilis*) in Fig. 5 (b). Fungi (*Aspergillus niger* and *Candida albicans*) were studied in Agar well-diffusion tests, which gave inhibition circles of between 14 and 21 mm, with *E.coli* and *A.niger* being the most sensitive. ZnO exhibited a definite dose-sensitive antibacterial activity in all the pathogens tested. The greatest sensitivity was recorded with Bacillus subtilis, where the inhibition zone was found to rise between 17.6 mm (25 ug/mL) and 26.64 mm (75 ug/mL), which is near to the positive control (32 mm). There was moderate susceptibility in E. coli, which increased with concentration to 11.6 mm up to 15.6 mm. In *Pseudomonas aeruginosa* and *Salmonella typhi*, there was a strong inhibitory effect over the concentration (29.6–32.6 mm), and this showed that the bacteria are highly sensitive to low doses.

**Fig. 6:**
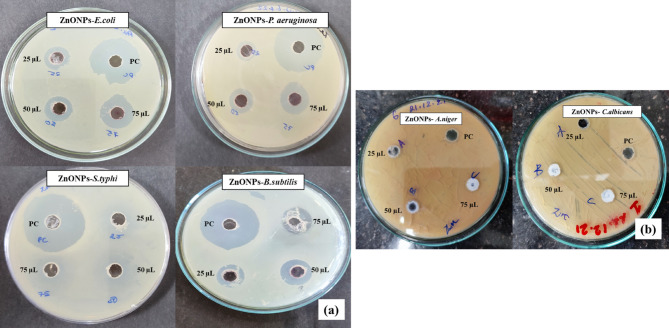
(**a**) Antibacterial and (**b**) Antifungal of ZnONPs.

The antifungal index was more than 65.5%, which indicated a wide-range antimicrobial action of the nanoparticles. Electrostatic Interaction: The ZnONP surface, with a potential of -26.8 mV, interacts with negatively charged bacterial membranes, thereby interfering with membrane integrity. The antifungal reaction was of the same tendency. *Aspergillus niger* showed an inhibition of 11, 16, and 21 mm at 25, 50 and 75 µL, respectively, and Candida albicans exhibited an inhibition of 9, 13, and 18 mm at 25, 50 and 75 µL, respectively. Zone of inhibition is seen in Fig. 6 (a) and (b) for bacterial and fungal activity. The growing inhibition is an indication of an augmented ROS-based membrane disruption under the higher nanoparticle concentrations.

Generation of reactive oxygen species (ROS), where photoexcitation of the ZnO nanoparticles results in the generation of superoxide and hydroxyl radicals that can oxidise cellular constituents, tearing them apart, leading to lipid peroxidation. Metal ion release: the released Zn ^2+^ ions enter the microbial cell, bind with the thiol (-SH) functional groups of vital enzymes and proteins, which, in turn, causes disturbances in respiratory and replicative processes. DNA damage: Oxidative stress, which is caused by ROS, results in the fragmentation of microbial DNA, which leads to cell death. This increased antibacterial activity against Gram-negative bacteria would be attributed to the relatively thin peptidoglycan barriers that allow diffusion of Zn ^2+^. Their synergistic oxidative and ionic activity highlights the strong antimicrobial efficacy of ZnO nanoparticles produced by *P. acidus*, making them good candidates to be used as antimicrobial rights/opaques, packaging, and the medical field.

### MB Dye degradations: Photocatalysis

**Fig. 7:**
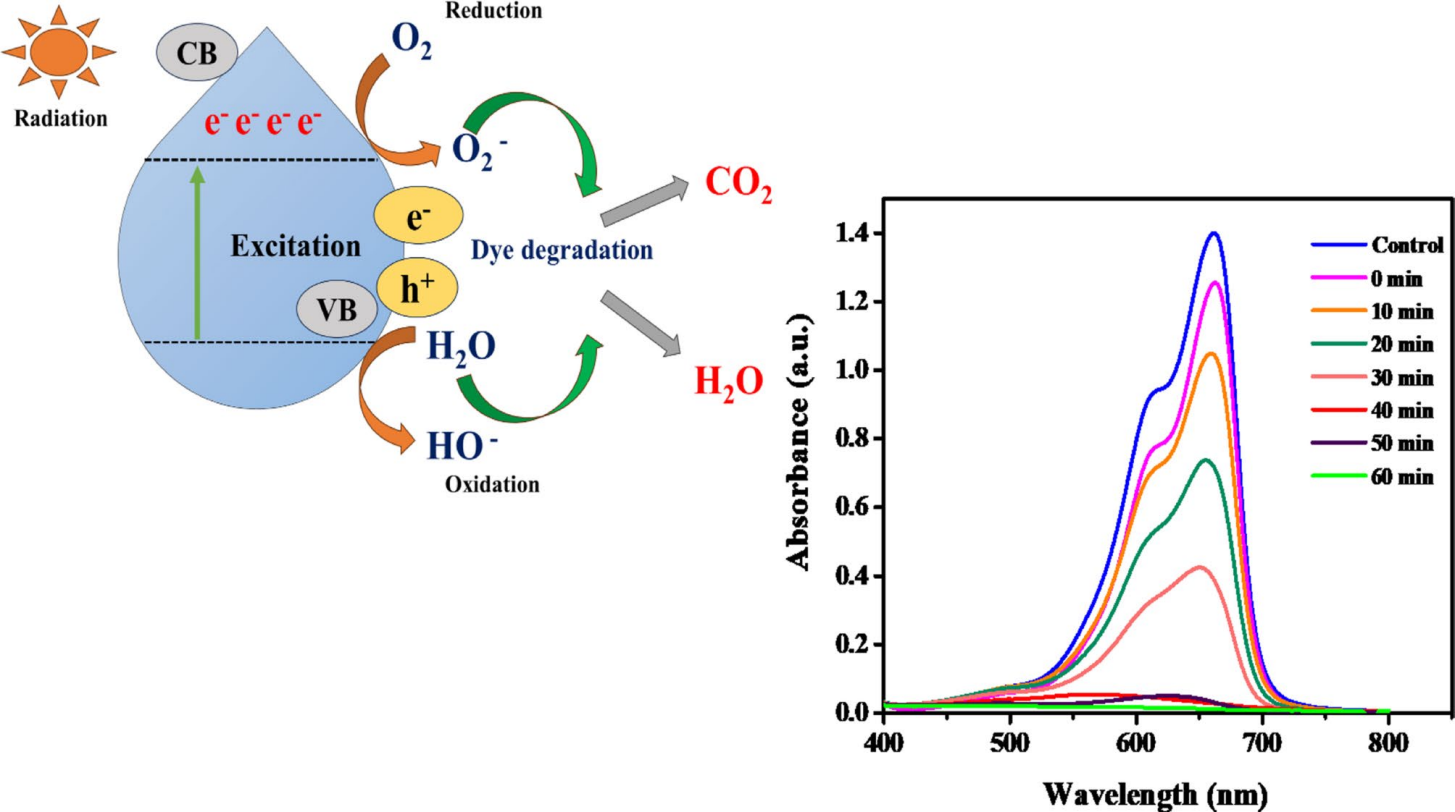
MB Dye degradation mechanism and graph of ZnONPs.

The photocatalytic activity of ZnONPs produced by *P. acidus* was determined by the degradation of MB dye under natural sunlight. The results obtained were the typical MB absorption peak at 664 nm, which faded in strength with the time of irradiation, demonstrating progressive degradation of dyes. The control experiments in the dark and without catalyst showed very low degradation (< 7% in 70 min), implying that the Methylene Blue removal is primarily controlled by ZnONPs-based photocatalytic reactions and not by photolysis and adsorption. When the optimal conditions are followed, ZnONP dose 50 mg/100 mL MB (10 mg/L), pH of 9.5 and ambient temperature, the nanoparticles degraded exceptionally to 99.8% in 70 min is seen in Fig. 7. The process of degradation had pseudo-first-order kinetics where the apparent rate constant (k) equal to 0.043 min^− 1^ was obtained when the equation was applied in a linearised form, ln(C_0_​/C_t​_) vs. time plot. Mechanistically, photoexcited electron-holes can be produced on ZnONPs on exposure to solar irradiation via:

7 $${\mathrm{ZnO}}+ {\mathrm{hv}}\to{\mathrm{e}}^{-}_{\mathrm{CB}} + {\mathrm{h}}^{+}_{\mathrm{VB}}$$

The electrons (e^−^) in the conduction band are then photogenerated to reduce the oxygen molecules that are adsorbed to superoxide radicals (O_2_^−^), with the holes (h^+^) in the valence band oxidising the oxygen molecules to hydroxyl radicals (OH). These reactive oxygen species (ROS) are powerful oxidants that oxidise MB by making it go through a sequence of demethylation and aromatic ring breakage, eventually leading to the formation of CO_2_ and H_2_O.

8 $$\:\mathrm{O}_\mathrm{2}+{\mathrm{e}_\mathrm{CB}}^{-}\to\:\mathrm{O}_{2}^{\mathrm{•}-},\mathrm{h}^{+}_\mathrm{VB}+\mathrm{H}_{2}\mathrm{O}\to\mathrm{•}\mathrm{OH}+\mathrm{H}^{+}$$

9 $$\:\mathrm{O}_{2}^{\mathrm{•}-},\mathrm{•}\mathrm{OH}+\mathrm{MB}\to\:{\mathrm{CO}}_{2}+{\mathrm{H}}_{2}\mathrm{O}$$

This is due to the synergistic action among the small particle size, high crystallinity and organic surface passivation that lowers the electron-hole recombination rate, resulting in a high photodegradation rate. ZnONP residual hydroxyl groups on its surface serve as anchors where MB is adsorbed, enhancing a high charge transfer efficiency in photocatalysis. The degradation kinetics were further confirmed by plotting the experimental results according to the pseudo-first-order kinetic equation, given by:

10 $$\:\mathrm{ln}\left(\frac{\mathrm{C}_{0}}{\mathrm{C}_\mathrm{t}}\right)=kt$$

Equation (10), A good linear fit was obtained from the plot of ln(C₀/Cₜ) versus time with a regression coefficient (R² ≈ 0.984), suggesting that the MB degradation process follows first-order kinetics. The value of R² suggests a good fit between the experimental and model data and shows that the rate of degradation of MB is governed by surface-based photocatalytic processes on ZnONPs.

### Photocatalytic stability: Reusability

**Fig. 8:**
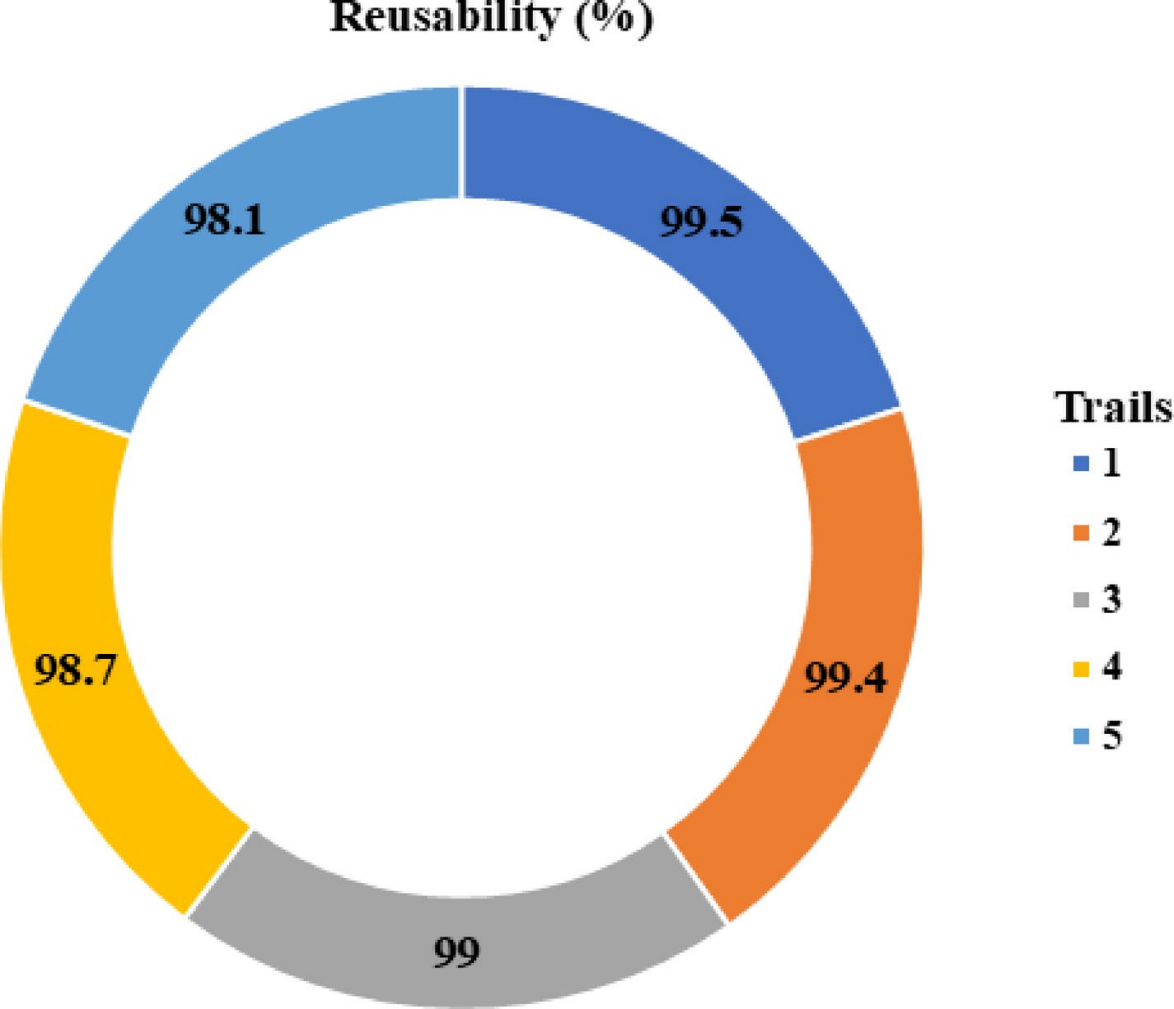
MB Dye degradation recyclability of ZnONPs.

The technical use of environmental technologies depends on such parameters as sustainability and recyclability. In this respect, the recyclability of ZnONPs was studied with the help of *P. acidus* in the course of 5 consecutive degradation cycles in the same experimental conditions. After this cycle, the photocatalyst was centrifuged, recovered, washed and reused with no observable structural changes in the photocatalyst. The degradation efficiency in the first and second cycles remained practically the same and amounted to 99.5 of 99.4%. The values of these values show that the surface-active sites and crystalline structure of the photocatalyst were not significantly altered after multiple exposures to the dye molecules and natural light. There was a slight decrease between the third and the fifth cycle, with efficiencies of 99, 98.7% Framework, and 98.1%, respectively, as noticed in Fig. 8.

The minor reduction is expected and can be caused by partial surface contamination or by adsorption of transitional degradation products to the active sites. Notably, the catalytic activity remained above the 98% mark after the fifth run, proving that the nanoparticles do not lose a significant proportion of their chemical activity and do not experience a significant structural loss. Through the post-reaction X-ray diffraction, it was realised that the wurtzite lattice was maintained, and there were no observable changes in the diffraction peaks, hence supporting the phase stability of the material. Table 1. The comparison between different dyes and photocatalysts is discussed. The ZnONPs were reclaimed after each run through centrifugation and washing, with very little loss (< 5 wt%) of the material; however, the photocatalytic activity still remains above 99%, suggesting that the activity is maintained despite the minute loss in weight. The slight reduction of degradation performance with each cycle of application proves the strength of the biogenic capping layer and indicates that the products of *P. acidus* transform ZnONPs, which double as long-term and reusable photocatalysts in biological photocatalysis of wastewater.

**Table 1:** Photocatalyst, targeted dye, light sources, initial dye concentration, and degradation efficiency are detailed.

Photocatalyst	Target Dye	Light Source	Initial Dye Concentration (ppm)	Catalyst Dose / Dye Volume (mg/mL)	Exposure Duration (min)	Degradation Efficiency (%)	Reference
ZnONPs (*Phyllanthus acidus* leaf extract)	Methylene Blue	Natural sunlight	10	100	70	99.8	*This work*
ZnO–Ag nanocomposite (*Phyllanthus emblica* extract)	Methylene Blue	Natural sunlight	10	100	70	99.7	^27^
ZnO NPs (*Moringa oleifera* leaf extract)	Methylene Blue	Natural sunlight	15	100	90	98.4	^28^
Fe₂O₃–TiO₂ nanocomposite (*Curcuma longa* extract)	Crystal Violet	UV–Vis light	15	50	110	98.1	^29^
Ag–TiO₂ nanocomposite (*Citrus limon* peel extract)	Direct Blue 6	Xenon lamp (500 W)	25	80	180	97.9	^30^
CuO NPs (*Punica granatum* peel extract)	Congo Red	Xenon lamp (300 W)	25	100	150	97.8	^31^
NiO–ZnO hybrid NPs (*Ocimum sanctum* extract)	Rhodamine B	White LED light	10	100	90	97.3	^32^
Co₃O₄ NPs (*Eucalyptus globulus* extract)	Reactive Black 5	Visible light	10	50	120	97.4	^33^
CuFe₂O₄ NPs (*Lawsonia inermis* extract)	Rhodamine B	Visible light	20	100	150	96.7	^34^
CeO₂ NPs (*Camellia sinensis* extract)	Methyl Orange	Visible light (500 W)	20	80	120	96.5	^35^
TiO₂ NPs (*Aloe vera* gel extract)	Rhodamine B	UV (365 nm)	20	50	120	96.2	^36^
ZnFe₂O₄ NPs (*Tridax procumbens* extract)	Congo Red	Sunlight	15	50	90	95.2	^37^
MnO₂ NPs (*Mentha piperita* extract)	Safranin O	UV light (254 nm)	30	100	180	94.6	^38^
Fe₃O₄ NPs (*Azadirachta indica* extract)	Malachite Green	Visible light	10	75	180	94.5	^39^

### Seed germinations

**Fig. 9:**
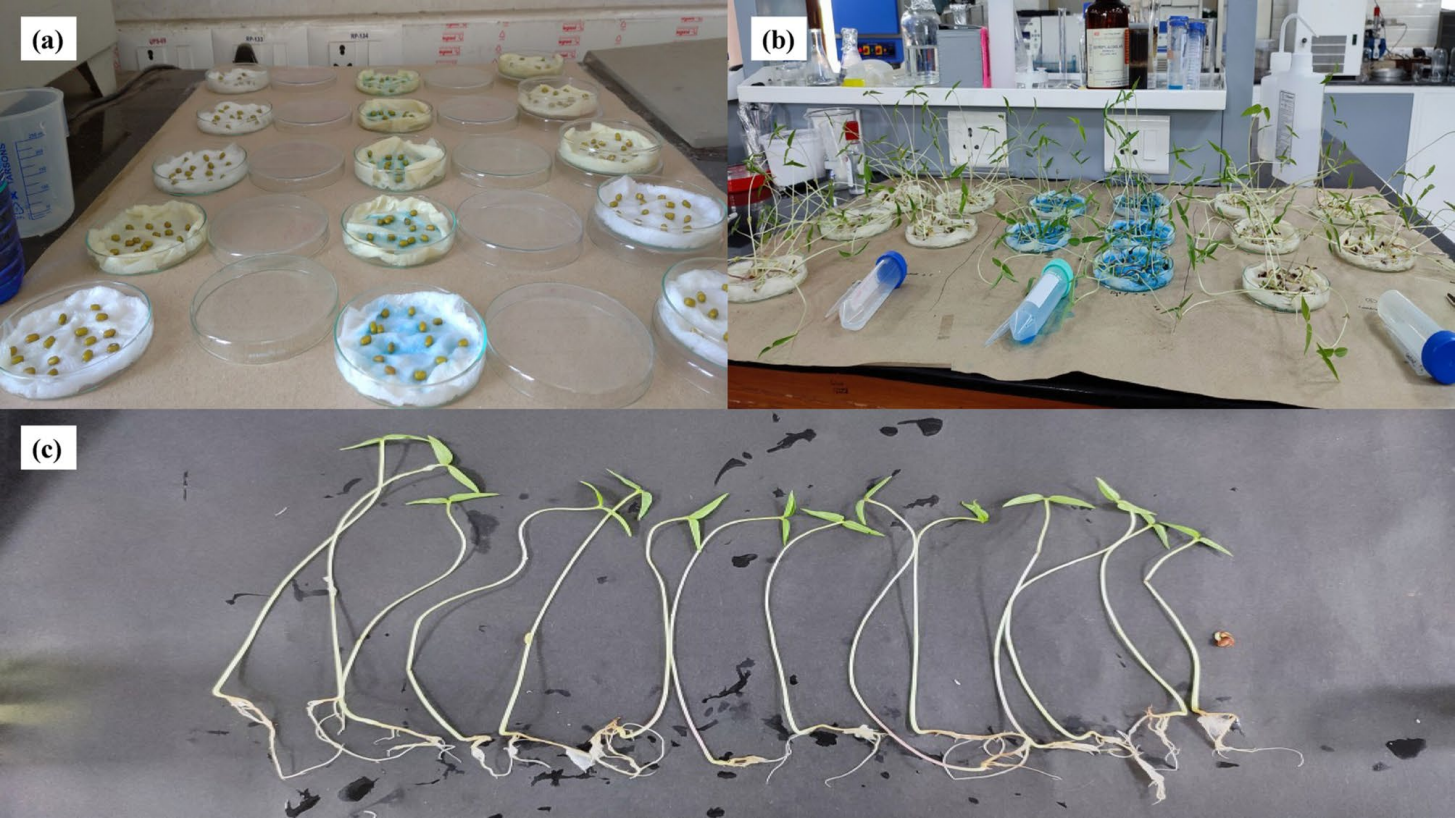
Seed germination of MB dye-treated water (**a**) Day 1, (**b**) Day 7, and (**c**) Length measurement.

An experiment of germination was performed on *Vigna radiata* to determine the phytotoxicity of an MB dye following the photocatalytic breakdown of the compound by ZnONPs derived through the *P.acidus*. The results showed that those seeds, through irrigation with ZnONPs-treated effluent, showed a significant increase in percentage of germination, increased velvety radical and plumule elongation and a high index of seedling vigour when compared to the control group that was irrigated with untreated dye solution. These findings prove that the photocatalytic pathway was effective in neutralising the toxic aromatic intermediates of MB to give benign mineral end-products. Figure 9 (a) indicates the day 1 of growth, (b) indicates the day 14 of the growth, and (c) gives the seed growth measurements. The measure of traces of bioavailable zinc to be released by the nanoparticles served as an important micronutrient, which triggers the activities of enzymes, chlorophyll synthesis, and protein metabolism, among the essential physiological functions that promote the early growth of the seedling. Such improved performance in the use of *P. acidus*-derived ZnONPs implies that it is an efficient photocatalyst to remove dyes and has agronomic characteristics, including nutrient supplement. Separate control studies with ZnONPs demonstrated negligible impacts on plant growth, confirming that the observed increase in plant growth is a result of the detoxification of the dye rather than the effects of the micronutrient, while statistical analysis (*p* < 0.001) confirms that the dye-containing effluent is non-toxic.

#### Statistical validation

Table 2 revealed significant physiological variation in the germination assay of *V. radiata* at the different irrigation conditions of control water, untreated MB effluent, and ZnONPs-treated dye water. It was evident in the analysis that ZnONPs-treated effluent was very strong in increasing seedling vigour, and untreated MB effluent was very stringent in exerting growth inhibition through all morphometric indices. There was a significant difference in the aerial morphology of *V. radiata* across the treatments. The average shoot length of untreated MB was 13.38 ± 5.26 cm, which is a 12.7% decrease from the control (15.33 ± 3.79), showing cell elongation was inhibited by dye toxicity. Notably, the mean shoot length of irrigation with ZnONPs-treated effluent was 17.98 ± 5.00 cm, which is an approximate 17.3% greater than the control and a 34% recovery relative to the MB-stressed seedlings.

**Table 2:** Descriptive statistics of seed germination parameters (Mean ± SD).

Parameter	Treatment	*N*	Mean ± SD
Shoot length (cm)	Control	62	15.33 ± 3.79
	MB Dye	59	13.38 ± 5.26
	Dye-treated water	73	17.98 ± 5.00
Root length (cm)	Control	62	1.58 ± 1.32
	MB Dye	59	1.95 ± 1.10
	Dye-treated water	73	3.15 ± 1.37
Shoot width (mm)	Control	62	1.32 ± 0.64
	MB Dye	59	0.79 ± 0.50
	Dye-treated water	73	1.33 ± 0.71
Root width (mm)	Control	62	0.55 ± 0.40
	MB Dye	59	0.25 ± 0.21
	Dye-treated water	73	0.80 ± 0.54

Similarly, the shoot width assumed a similar pattern; the control (1.32 ± 0.64 mm) showed a significant difference with the MB group (0.79 ± 0.50 mm), but the limit of the contrast was a significant enhancement of zinc oxide nanoparticles in pigment-induced stress alleviation (1.33 ± 0.71 mm). The increased biomass of the shoot is associated with higher synthesis of chlorophyll and a high photosynthetic efficiency because zinc plays a major role as an enzymatic cofactor in carbonic anhydrase and morphogenic pathways. Even stronger differentiation was indicated by root parameters. Root elongation at MB dye exposure was limited to 1.95 ± 1.10 cm, which is almost half that in the ZnONPs-treated condition (3.15 ± 1.37 cm). The intermediate root growth of control seedlings (1.58 ± 1.32 cm) indicated that although the presence of MB stress inhibited the growth of root length, the products of degradation facilitated by nanoparticles and the accumulation of zinc in the seedling, altogether reinstated rhizogenic functions.

The root width parameter was the most sensitive to changes in treatment. The MB-treated roots established an average diameter of 0.25 ± 0.21 mm, which is a sign of damage to the cell wall and non-differentiation of the vascularity under dye toxicity. ZnONPs-treated irrigation resulted in roots with a much greater mean width (0.80 ± 0.54 mm), which was 3.2 times an improved result over MB dye exposure and 45% over the control conditions. Such morphological recovery indicates the maintenance of the osmotic stability, ion exchange capacity and the metabolic activity with the help of the ZnONPs-mediated effluent detoxification. As demonstrated in Table 3, the one-way ANOVA data showed that the difference between treatments of all the measured parameters was very significant (*p* < 0.001). Root characteristics showed the highest F-statistical value, root length (F = 28.40) and root width (F = 27.99): this indicates that the root area is the most sensitive physiological area to the ZnONPs-treated irrigation. In the same way, there was a great variance in the shoot parameters (F = 15.77 with shoot length; F = 14.30 with shoot width), confirming the reproducibility of the growth enhancement effect.

**Table 3:** One-way ANOVA for seed germination parameters.

Parameter	F-statistic	*p*-value	Significance
Shoot length (cm)	15.77	4.57 × 10⁻⁷	Significant
Root length (cm)	28.40	1.59 × 10⁻¹¹	Significant
Shoot width (mm)	14.30	1.63 × 10⁻⁶	Significant
Root width (mm)	27.99	2.18 × 10⁻¹¹	Significant

#### Scientific implications

Photocatalytic degradation of MB dye, which changes the toxic aromatic intermediates to the less toxic or mineralised forms like CO_2_, H_2_O and nitrates, thus removing phytotoxic stressors; Slow dissociation of the Zn^2+^ ions in the nanoparticle matrix that serves as micronutrients that promote chlorophyll biosynthesis, enzymatic activation, and auxin production that contributes to meristematic activity. The increased root thickness and shoot growth suggest increases in nutrient uptake and cellular division rates, indicating that the treated nanoparticle-treated industrial effluents not only lose toxicity but also become biostimulatory, which is in line with sustainable agriculture and green remediation. In general, the results of ANOVA and the growth parameters scientifically prove that ZnONPs-treated water markedly improves germination and early development of plants, which justifies its scientific usefulness as an eco-restorative and agronomically efficient nanomaterial.

## Conclusion

The ZnONPs with high purity, high stability, and multifunctionality were obtained through the green synthesis of ZnONPs using the *P. acidus* plant. A strong absorption band at 344 nm, an average hydrodynamic diameter of 21. 36 nm and thermal stability at 255℃ confirmed the wurtzite crystal phase. The photocatalytic experiments showed that the decomposition of methylene blue had an efficiency of 99.8% with the production of benign intermediates that, in turn, facilitated seed germination of the green gram as compared to the aqueous controls, and this enhancement was probably. Based on this, the goal of this study was to create a sustainable, non-toxic ZnO nanomaterial that lies between green synthesis and real multifunctional reuse. The joint measurement of photocatalytic activity and biological efficacy showed that ZnONPs produced by *P. acidus* retained their structure and exhibited the best antioxidant and antimicrobial properties. The novelty of the work is observed in the correlation of the eco-friendly synthesis, the environmental purification, the bio-stimulation, and thus making these nanoparticles reusable and nutrient-enriched catalysts with potential purposes in wastewater treatment and sustainable agriculture.

## Data Availability

The data used to support the findings of this study are included in the article. Should further data or information be required, these are available from the corresponding author (M. Shanmugavel) upon request.
